# Psychosocial Factors, Self-Care and Medication Adherence among Individuals Living with Hypertension in Greece: A Study Protocol

**DOI:** 10.1192/j.eurpsy.2026.11031

**Published:** 2026-07-31

**Authors:** T. Stamoulis, Z. Konstanti, E. Laiou, M. Gouva, M. Kourakos

**Affiliations:** Scientific Laboratory of Psychology and Person-Centered Care, Department of Nursing, School of Health Sciences, University of Ioannina, Ioannina, Greece

## Abstract

**Introduction:**

Hypertension is a major chronic health issue affecting individuals in all parts of the world, with psychosocial factors playing a key role in adherence to treatment and engagement in essential self-care behaviors. Personality traits and perceived quality of life may influence these processes, making it essential to understand these associations for the development of personalized, psychologically informed interventions.

**Objectives:**

This study aims to investigate the psychosocial profile of adults diagnosed with hypertension in Greece and examine its relationship with self-care behaviors and medication adherenceSpecifically, the study will explore the associations between personality traits, quality of life, self-care behaviors, and adherence to antihypertensive treatment.

**Methods:**

This is a cross-sectional, observational study currently underway, with data collection having commenced in early 2025. Data are being collected from a convenience sample of individuals with elevated blood pressure in primary care settings across Greece. The study instruments include: a demographic questionnaire, the SF-36 Health Survey (for quality of life assessment), the Eysenck Personality Questionnaire (EPQ) (for personality traits), the Self-Care of Hypertension Inventory, version 3 (SC-HI v3), and the 8-item Morisky Medication Adherence Scale (MMAS-8). Descriptive and inferential statistics are being employed to examine the associations between psychological factors, self-care, and medication adherence.

**Results:**

It is expected that certain personality profiles (e.g., high neuroticism, low conscientiousness) and lower levels of perceived quality of life will be associated with reduced adherence to antihypertensive therapy and lower self-care engagement. These findings align with existing literature, suggesting that psychosocial factors significantly influence treatment adherence and self-care practices among people living with hypertension. The results will help inform clinical practice by highlighting the importance of incorporating psychosocial assessments into routine hypertension management.

**Conclusions:**

This protocol outlines a planned study examining the intersection of personality, quality of life, self-care, and medication adherence in people in Greece who are managing hypertension. By identifying psychological and behavioral patterns, the study seeks to provide a foundation for targeted psychosocial interventions aimed at improving outcomes for individuals living with elevated blood pressure.

**Disclosure of Interest:**

None Declared

